# Urine Soluble CD163 Is a Promising Biomarker for the Diagnosis and Evaluation of Lupus Nephritis

**DOI:** 10.3389/fimmu.2022.935700

**Published:** 2022-07-14

**Authors:** Yun-Ju Huang, Chiung-Hung Lin, Huang-Yu Yang, Shue-Fen Luo, Chang-Fu Kuo

**Affiliations:** ^1^ School of Medicine, Chang Gung University, Taoyuan City, Taiwan; ^2^ Division of Rheumatology, Allergy and Immunology, Chang Gung Memorial Hospital, Taoyuan City, Taiwan; ^3^ Division of Thoracic medicine, Chang Gung Memorial Hospital, Taoyuan City, Taiwan; ^4^ Division of Nephrology, Allergy and Immunology, Chang Gung Memorial Hospital, Taoyuan City, Taiwan; ^5^ Center for Artificial Intelligence in Medicine, Chang Gung Memorial Hospital, Taoyuan, Taiwan

**Keywords:** Systemic lupus erythematosus, lupus nephritis, urine soluble CD163, urine biomarker, chronic kidney disease, macrophage, SLEDA

## Abstract

**Introduction:**

Urine-soluble CD163 (usCD163) is released from alternatively activated macrophages involved in the resolution of inflammation in glomeruli and plays an important role in glomerulonephritis. This study explored the role of usCD163 in patients with systemic lupus erythematosus (SLE).

**Materials and Methods:**

usCD163 concentrations were measured cross-sectionally in 261 SLE patients in Taiwan. Clinical and laboratory data were collected, and SLE disease activity scores were calculated to assess the correlation with usCD163.

**Results:**

SLE patients with high usCD163 levels tended to be younger, with a higher hospital admission rate, higher prednisolone dose, lower estimated glomerular filtration rate, higher urine protein creatinine ratio (UPCR), more pyuria and hematuria, higher levels of inflammatory markers, higher rates of anemia, neutropenia, and lymphopenia, lower complement 3 (C3) levels, higher anti-double-stranded DNA antibody (anti-dsDNA Ab) levels, and higher disease activity scores (p < 0.05). usCD163 levels were significantly higher in patients with active lupus nephritis (LN) than in those with extrarenal or inactive SLE and correlated with UPCR, disease activity, and anti-dsDNA Ab levels. SLE patients with high usCD163 levels tended to have a higher chronic kidney disease stage.

**Discussion and conclusion:**

The usCD163 level correlates with the severity of LN and disease activity in renal SLE.

## Introduction

CD163 is a 130-kDa type I glycosylated transmembrane protein belonging to the cysteine-rich scavenger receptor superfamily type B that can bind the hemoglobin–haptoglobin complex during intravascular hemolysis, both physiologically and pathologically (1, 2). CD163-positive macrophages are alternatively activated M2 macrophages exhibiting an anti-inflammatory function and contribute to wound healing (3). CD163-positive macrophages are found in several human glomerular diseases, such as poststreptococcal glomerulonephritis, ANCA-associated vasculitis, diabetic nephropathy, and lupus nephritis (LN) (4, 5). Soluble CD163 is derived from the cleavage of the CD163 macrophage receptor by metalloproteinase and is released into inflammatory tissue, blood, urine, cerebrospinal fluid, and synovial fluid (6) during systemic inflammatory responses such as sepsis, liver disease, malaria, autoimmune disorders, and reactive hemophagocytic syndrome (7). Serum CD163 concentrations in peripheral blood reflect the disease severity in autoimmune diseases such as rheumatoid arthritis, systemic sclerosis, and idiopathic inflammatory myositis (6, 8). However, urine-soluble CD163, but not serum CD163, correlated with the severity of LN in some studies- (4, 9).

Serious complications of systemic lupus erythematosus (SLE) include hemolytic anemia, refractory severe thrombocytopenia, cardiac tamponade, massive bilateral pleural effusions, central nervous system lupus, macrophage activation syndrome, and proliferative LN (10, 11). More than half of SLE patients have renal disease, with the high risk of renal function deterioration and mortality, particularly patients who present with proliferative glomerular disease composed of the renal histology of International Society of Nephrology/Renal Pathology Society (ISN/RPS) class III/VI (12, 13). Approximately 10%–17% of LN patients will progress to end-stage renal disease, despite immunosuppressants (14). Markers of SLE activity, including serum complement 3 (C3), C4, and anti-double stranded DNA antibody (anti-dsDNA Ab), are conventional biomarkers for assessing disease severity (15) but lack sufficient sensitivity and specificity for histological classification of LN (16). Renal biopsy is the gold standard for histological assessment of LN but has shortcomings in terms of sampling error, invasiveness, and the complication of hemorrhage (17). An alternative non-invasive and reproducible biomarker, such as urine CD163, is needed for evaluating LN (18, 19). Specific urinary biomarkers that discriminate active LN could be instrumental in guiding its management (9, 20).

In this study, we evaluated usCD163 as a potential biomarker for LN and investigated its correlation with clinical characteristics, laboratory parameters, and disease activity, thereby providing a theoretical basis for novel therapeutics for LN.

## Materials and methods

### Patients, Sample Collection, and Preparation

This cross-sectional study was performed to evaluate usCD163 as a potential biomarker of LN disease activity in Taiwan. The study was approved by the Institutional Review Board of Chang Gung Memorial Hospital, and informed consent was obtained from all patients. Blood and urine samples were collected from 261 patients with SLE on admission to Chang Gung Memorial Hospital and its affiliated hospitals between January 2019 and September 2021. All patients met the 1997 American College of Rheumatology revised criteria (21) or 2012 Systemic Lupus International Collaborating Clinics (SLICC) classification criteria (22) for SLE. There was no severe acute respiratory syndrome coronavirus 2 (SARS-CoV-2) infection among our SLE cohort.

The Systemic Lupus Erythematosus Disease Activity Index 2000 (SLEDAI-2K) covers seizures, psychosis, organic brain syndromes, visual disturbance, cranial nerve disorder, lupus headache, cerebrovascular accident, vasculitis, urinary casts, hematuria, proteinuria, pyuria, pleurisy, pericarditis, and fever (23). The renal SLEDAI (rSLEDAI) provides a sum score for the renal domains of SLEDAI, including hematuria (>5 red blood cells/high-power field), pyuria (>5 white blood cells/high-power field), proteinuria (>0.5 g/24 h), and urinary casts. SLE patients were classified into three groups: rSLEDAI score ≥4 (active LN), SLEDAI-2k score ≥5 (active extrarenal SLE), and rSLEDAI score = 0. The inactive (or low-disease activity) group included SLE patients with a SLEDAI-2k score ≤ 4, and the clinical SLEDAI (omitting anti-dsDNA and complement) included those with a SLEDAI-2k score ≤ 2.

SLEDAI, rSLEDAI, complete blood count, blood urea nitrogen, serum creatinine, urinalysis, urine protein creatinine ratio (UPCR), C3, C4, and anti-dsDNA data were collected. LN histology was determined based on the ISN/RPS classification, and histological scores (evaluated according to acute and chronic indices) were recorded. The Modification of Diet in Renal Disease formula was used to calculate the estimated glomerular filtration rate (eGFR, as follows:

$eGFR=186\times {serum\;creatinine}^{-1.151}\times {age}^{-0.203}(\times 0.742\;\;$ *in females*). The results are expressed as mL/min/1.73 m^2^.

### UsCD163 Data Collection, Preparation, and Analysis

Clean-catch midstream urine samples were collected and refrigerated within 1 h of collection. The samples were then aliquoted and stored at -80°C. To detect usCD163, an enzyme-linked immunosorbent assay (ELISA) was performed using a human soluble CD163 ELISA Kit (Euroimmun, Lübeck, Germany) in accordance with the manufacturer’s instructions. The lower detection limit of the sCD163 ELISA is 0.15 ng/ml. A cutoff for usCD163 positivity of 1.27 ng/ml was used, or a normalized cutoff of 242.30 ng/mmol creatinine that was referenced from the instruction brochure from Euroimmun.

### Statistical Analyses

Count data are expressed as frequencies and rates, and comparisons of rates were done using the cube test. Normally distributed data are expressed as the mean and standard deviation. The t-test was used for group comparisons, and Pearson’s correlation analysis was also performed. Continuous variables were compared using Welch’s t-test. The coefficient of determination was taken to indicate the strength of the association between two continuous variables. The chi-squared and Fisher’s exact tests were used for comparing two groups, and analysis of variance was used for comparing multiple groups, with *post-hoc* pairwise comparisons. The non-parametric Spearman correlation test was also performed. All data are presented as box plots, where the upper whiskers represent the 75th percentile + 1.5 × the interquartile range (IQR), and the lower whiskers represent the 25th percentile - 1.5 × IQR. IBM SPSS Statistics for Windows software (version 25.0; IBM Corp., Armonk, NY, USA) was used for data processing, and p < 0.05 was considered to indicate a statistically significant difference.

## Results

### Patient Characteristics

The 261 SLE patients in Taiwan had an average age of 44.4 years, and women predominated (95.4%) (**Table 1**). Over half of the patients were taking prednisolone (79%, 9 mg/day) and/or hydroxychloroquine (69%, 266 mg/day). The average creatinine level was 0.8 mg/dl, the eGFR was 100 ml/min/1.73 m^2^, and the UPCR was 920 mg protein/g creatinine. The average usCD163 level was 2.8 ng/ml, and the urine usCD163/creatinine ratio was 842 ng/mmol. The average SLEDAI-2K score was 7.7 points in those with a high anti-ds DNA Ab level (170 IU/ml), low C3 level (81 mg/dl), orC4 (15 mg/dl) level.

**Table 1:** Patient characteristics (n = 261 SLE patients).

	Average ± standard deviation (max, min)
Age (years)	44.4 ± 11.8 (88,19)
Sex (male: female)	12:249
Body weight (kg)	57.4 ± 11.5 (132,35)
Body height (cm)	157.9 ± 6.5 (184,130)
BMI (kg/m^2^)	23 ± 4.5 (57.9,14)
Admission, n (%)	24 (9.2%)
Diabetes mellitus, n (%)	14 (5.4%)
Macrophage activation syndrome, n (%)	2 (0.8%)
CNS lupus, n (%)	1 (0.4%)
Infection, n (%)	8 (3.1%)
Prednisolone, n (%)	208 (79.7%), average dose 9mg/day
Hydroxychloroquine, n (%)	182 (69.7%), average dose 266.5mg/day
Mycophenolate mofetil, n (%)	16 (6.1%), average dose 1077.5 mg/day
Mycophenolate sodium, n (%)	57 (21.8%), average dose 703.8mg/day
Azathioprine, n (%)	30 (11.5%), average dose 53.3mg/day
Cyclosporine, n (%)	11 (4.2%), average dose 109 mg/day
Methotrexate, n (%)	12 (2.7%), average dose 9.8 mg/week
Pulse steroid, n (%)	5 (1.9%), average dose 3g
Serum creatinine (mg/dL)	0.8 ± 0.7 (7,0)
BUN (mmol/L)	30.3 ± 37.2 (297,5)
EGFR (mL/min/1.73 m^2^)	100.8 ± 48.8 (391,0)
UPCR (mg protein/g creatinine)	920.1 ± 2102.6 (18393,0)
Semiquantitative proteinuria in urinary dipstick	Trace:23 (8.8%) 1+:27 (10.3%) 2+:31 (11.9%) 3+:26 (10%) 4+:8 (3.1%)
Semiquantitative hematuria in urinary dipstick	Trace: 18 (6.9%) 1+:25 (9.6%) 2+:26 (10%) 3+:21 (8%)
RBC in urinary dipstick (HPF)	22.2 ± 67.9 (500,0)
WBC in urinary dipstick (HPF)	29.1 ± 69.3 (500,0)
CRP (mg/L)	5 ± 11.1 (82,0)
ESR (mm/h)	24.7 ± 20.3 (140,1)
WBC (/µL)	6.4 ± 3 (20,2)
Hb (g/dL)	12.2 ± 1.8 (19,7)
Neutrophil (/µL)	4656.8 ± 2834.8 (19000,748)
Lymphocyte (/µL)	1337.8 ± 727.2 (4572,78.3)
Platelet (1,000/µL)	232.7 ± 89.1 (547,5)
C3 (mg/dL)	81.1 ± 24.4 (173,18)
C4 (mg/dL)	15.2 ± 8.1 (41,2)
Anti-ds DNA Ab (IU/mL)	170.7 ± 162.6 (648,14)
Pleural effusion, n (%)	10 (3.8%)
Pericardial effusion, n (%)	4 (1.5%)
SLEDAI-2K, points	7.7 ± 5 (27,0)
r-SLEDAI, points	4.7 ± 4.1 (12,0)
Inactive SLE, n (%) Extrarenal SLE, n (%) Renal SLE, n (%)	156 (59.8%) 9 (3.4%) 96 (36.8%)
usCD163 (ng/mL)	2.8 ± 9.3 (96.3,0)
High usCD163, n (%)	63 (25.1%)
usCD163/creatinine in urine (ng/mmol)	366.5 ± 870.8 (6926.8,13.2)
High usCD163/creatinine in urine, n (%)	69 (26.4%)

BMI, body mass index; CNS, central nervous system; BUN, blood urea nitrogen; EGFR, estimated glomerular filtration rate; UPCR, urine protein creatinine ratio; RBC, red blood cell; WBC, white blood cell; CRP, C-reactive protein; ESR, erythrocyte sedimentation rate; Hb, hemoglobin; C3, complement 3; C4, complement 4; anti-ds DNA Ab, anti-double-strand DNA antibody; SLEDAI-2k, Systemic Lupus Erythematosus Disease Activity Index 2000; r-SLEDAI, Renal Systemic Lupus Erythematosus Disease Activity Index.

### Clinical and Laboratory SLE Data of Patients With and Without High usCD163 Levels

Patients were divided into a high usCD163 group (n = 63, average usCD163 level = 10 ng/ml) and low usCD163 group (n = 198, average usCD163 level = 0.5 ng/ml) (**Table 2**). The former group of patients tended to be younger and had a higher hospital admission rate, mycophenolate sodium usage, and prednisolone dose (p < 0.05). They also had a lower eGFR, higher UPCR, higher rates of pyuria, hematuria, anemia, neutropenia, and lymphopenia, higher inflammatory marker levels, lower C3 levels, higher anti-dsDNA Ab levels, higher SLEDAI-2K scores, and higher urine usCD163/creatinine ratio (p < 0.05). Six (9.5%) inactive SLE patients and no extrarenal SLE patients had high usCD163 levels. The histological results of the initial kidney biopsy for 43 LN patients, according to the ISN/RPS class and activity and chronicity indices, are shown in **Table 3**. The glomerular sclerosis of the chronicity index correlated with both high usCD163 and high usCD163/creatinine in urine with clinical significance.

**Table 2:** Clinical and laboratory data of SLE with low and highusCD163.

	Low usCD163 N = 198	High usCD163 N = 63	P value
Age (years)	45.4 ± 11.3	41.2 ± 13	0.013*
Sex (male: female)	10:188	2:61	0.414
BMI (kg/m^2^)	23.1 ± 4.4	22.8 ± 5	0.675
Admission, n (%)	12 (6.1%)	12 (19%)	0.004*
Diabetes mellitus, n (%)	11 (5.6%)	3 (4.8%)	1.000
Macrophage activation syndrome, n (%)	2 (1%)	0	0.575
CNS lupus, n (%)	1 (0.5%)	0	1.000
Infection, n (%)	5 (2.5%)	3 (4.8%)	0.299
Prednisolone, n (%)	154 (77.8%)	54 (85.7%)	0.116
	6.3 ± 7.7 mg/day	9.9 ± 8 mg/day	0.001*
Hydroxychloroquine, n (%)	137 (69.2%)	45 (71.4%)	0.433
	181.3 ± 145 mg/day	200 ± 152 mg/day	0.380
Mycophenolate mofetil, n (%)	12 (6.1%)	4 (6.3%)	0.568
Mycophenolate sodium, n (%)	29 (14.6%)	28 (44.4%)	<0.001*
Azathioprine, n (%)	21 (10.6%)	9 (14.3%)	0.277
Cyclosporine, n (%)	6 (3%)	5 (7.9%)	0.097
Methotrexate, n (%)	10 (5.1%)	2 (3.2%)	0.423
Pulse steroid, n (%)	2 (1%)	3 (4.8%)	0.093
Serum creatinine (mg/dL)	0.8 ± 0.7	1 ± 0.6	0.074
BUN (mmol/L)	23.9 ± 17.1	37.4 ± 50.5	0.123
EGFR (mL/min/1.73 m^2^)	105.3 ± 48.6	88.4 ± 45.9	0.017*
UPCR (mg protein/g creatinine)	315 ± 731.4	2715.9 ± 3430.6	<0.001*
Semiquantitative proteinuria in urinary dipstick	None:136 (79.1%) Trace: 18 (9.3%) 1+:23 (11.9%) 2+:13 (6.7%) 3+:14 (2.1%)	None:6 (9.5%) Trace: 5 (7.9%) 1+:4 (6.3%) 2+:18 (28.6%) 3+:22 (34.9%) 4+:8 (12.7%)	
Semiquantitative hematuria in urinary dipstick	None:141 (72.7%) Trace: 11 (5.7%) 1+:17 (8.8%) 2+:19 (9.8%) 3+:6 (3.1%)	None:26 (41.3%) Trace: 7 (11.1%) 1+:8 (12.7%) 2+:7 (11.1%) 3+:15 (23.8%)	
RBC in urinary dipstick (HPF)	14.6 ± 54.5	45.1 ± 94.8	0.018*
WBC in urinary dipstick (HPF)	22.6 ± 66.8	48.9 ± 73.3	0.009*
CRP (mg/L)	4.3 ± 9.4	7 ± 14.8	0.118
ESR(mm/h)	22.4 ± 19.4	32.3 ± 21.7	0.002*
WBC (/µL)	6.2 ± 2.8	7 ± 3.5	0.065
Hb (g/dL)	12.4 ± 1.7	11.3 ± 2.1	<0.001*
Neutrophil (/µL)	4279.2 ± 2562.9	5802.2 ± 3301.6	<0.001*
Lymphocyte (/µL)	1430.8 ± 686.1	1055.8 ± 780.1	<0.001*
Platelet (1,000/µL)	228.9 ± 81.4	244.3 ± 109.4	0.306
C3 (mg/dL)	83.6 ± 20.5	73.4 ± 32.8	0.023*
C4 (mg/dL)	15.5 ± 7.6	14.2 ± 9.4	0.277
Anti-ds DNA Ab (IU/mL)	150.3 ± 140.5	234.2 ± 206.4	0.003*
Pleural effusion, n (%)	7 (3.5%)	3 (4.8%)	0.449
Pericardial effusion, n (%)	0	1 (1.6%)	0.353
SLEDAI-2K, points	6.5 ± 4.3	11.7 ± 4.9	<0.001*
r-SLEDAI, points	3.5 ± 3.4	8.5 ± 3.5	<0.001*
usCD163 (ng/mL)	0.5 ± 0.2	10 ± 17.1	<0.001*
usCD163/creatinine in urine (ng/mmol)	126 ± 117.1	1032.1 ± 1497.5	<0.001*
High usCD163/creatinine in urine, n (%)	27 (17.4%)	42 (75%)	<0.001*
Renal biopsy	II:1 III:9 IV:16 V:6 III+V:1 IV+V:1	II:2 III:5 IV:14 V:2 III+V:4 IV+V:1	
Inactive SLE, n (%) Extrarenal SLE, n (%) Renal SLE, n (%)	Inactive SLE, 90 (45.5%) Extrarenal SLE, 9 (4.5%) Renal SLE, 99 (50%)	Inactive SLE, 6 (9.5%) Extrarenal SLE, 0 (0%) Renal SLE, 57 (90.5%)	<0.001*

BMI, body mass index; CNS, central nervous system; BUN, blood urea nitrogen; EGFR, estimated glomerular filtration rate; UPCR, urine protein creatinine ratio; RBC, red blood cell; WBC, white blood cell; CRP, C-reactive protein; ESR, erythrocyte sedimentation rate; Hb, hemoglobin; C3, complement 3; C4, complement 4; anti-ds DNA Ab, anti-double strand DNA antibody; SLEDAI-2k, Systemic Lupus Erythematosus Disease Activity Index 2000; r-SLEDAI, Renal Systemic Lupus Erythematosus Disease Activity Index. The meaning of bold values is that p <0.05 is significant.

**Table 3:** Histology of initial kidney biopsy in 43 lupus nephritis patients.

	Number of patients (%)	High usCD163, n (%)	P value	High usCD163/creatinine in urine, n (%)	P value
ISN/RPS class			0.386		0.339
II	3 (7%)	2 (66.7%)		3 (100%)	
III	10 (23.3%)	4 (40%)		2 (20%)	
IV	19 (44.2%)	14 (73.7%)		15 (78.9%)	
V	5 (11.6%)	2 (40%)		2 (40%)	
III+V	4 (9.3%)	4 (100%)		1 (25%)	
IV+V	2 (4.7%)	1 (50%)		2 (100%)	
Activity index (0–24)	6.6 ± 3.3 (13,1)	5.8 ± 2.7	0.337	7.1 ± 3.0	0.592
Endocapillary hypercellularity	22/24 (91.7%)	11/22 (50%)	0.482	11/22 (50%)	0.275
Glomerular leukocyte infiltration	12/24 (50%)	5/12 (41.7%)	0.275	7/12 (58.3%)	0.505
Wire loop deposits	8/24 (33%)	2/8 (25%)	0.082	2/8 (25%)	0.068
Fibrinoid necrosis	7/24 (29.2%)	2/7 (28.6%)	0.154	3/7 (42.9%)	0.552
Cellular crescents	15/24 (62.5%)	7/15 (46.7%)	0.358	8/15 (53.3%)	0.103
Interstitial inflammation	16/24 (66.7%)	9/16 (56.3%)	0.211	8/16 (50%)	0.385
Hyaline thrombi	2/24 (8.3%)	1/2 (50%)	0.642	1/2 (50%)	0.835
Chronicity index (0–12)	2.4 ± 2.3 (9,0)	2.6 ± 2.5	0.711	3.2 ± 2.6	0.213
Glomerular sclerosis	9/24 (37.5%)	8/9 (88.9%)	0.004*	8/9 (88.9%)	0.012*
Fibrous crescents	1/24 (4.2%)	1/1 (100%)	0.363	1/1 (100%)	0.666
Tubular atrophy	7/24 (29.2%)	5/7 (71.4%)	0.208	6/7 (85.7%)	0.127
Interstitial fibrosis	17/24 (70.8%)	7/17 (41.2%)	0.360	8/17 (47.1%)	0.728

ISN/RPS, International Society of Nephrology/Renal Pathology Society. The meaning of bold values is that p <0.05 is significant.

### Correlations of the usCD163 Level With the UPCR, SLEDAI2K Score, and Anti-Ds DNA Ab Level in SLE Patients

There were modest correlations with statistical significance of the usCD163 level with the UPCR, anti-dsDNA Ab level, and SLEDAI-2K and rSLEDAI scores in the SLE patients. Also, there were moderate correlations with statistical significance of the urine usCD163/creatinine ratio with the UPCR and SLEDAI-2K and rSLEDAI scores (**Table 4**). A usCD163 level of 0.443 ng/ml and a urine usCD163/creatinine ratio of 110.2 ng/mmol predicted high SLEDAI scores (more than six points) (**Supplementary Figure 1, 2**).

**Table 4:** Correlation between usCD163 and UPCR/anti-ds DNA Ab/SLEDAI/rSLEDAI in SLE patients; P < 0.0001.

R, coefficients of determination	UPCR (mg protein/g creatinine)	Anti-ds DNA Ab (IU/mL)	SLEDAI (points)	rSLEDAI (points)
usCD163 (ng/mL)	0.387	0.329	0.375	0.380
usCD163/creatinine (ng/mmol)	0.689	0.267	0.344	0.367

### Correlation between the usCD163 level and SLEDAI-2K score in SLE patients

**Figure 1:**
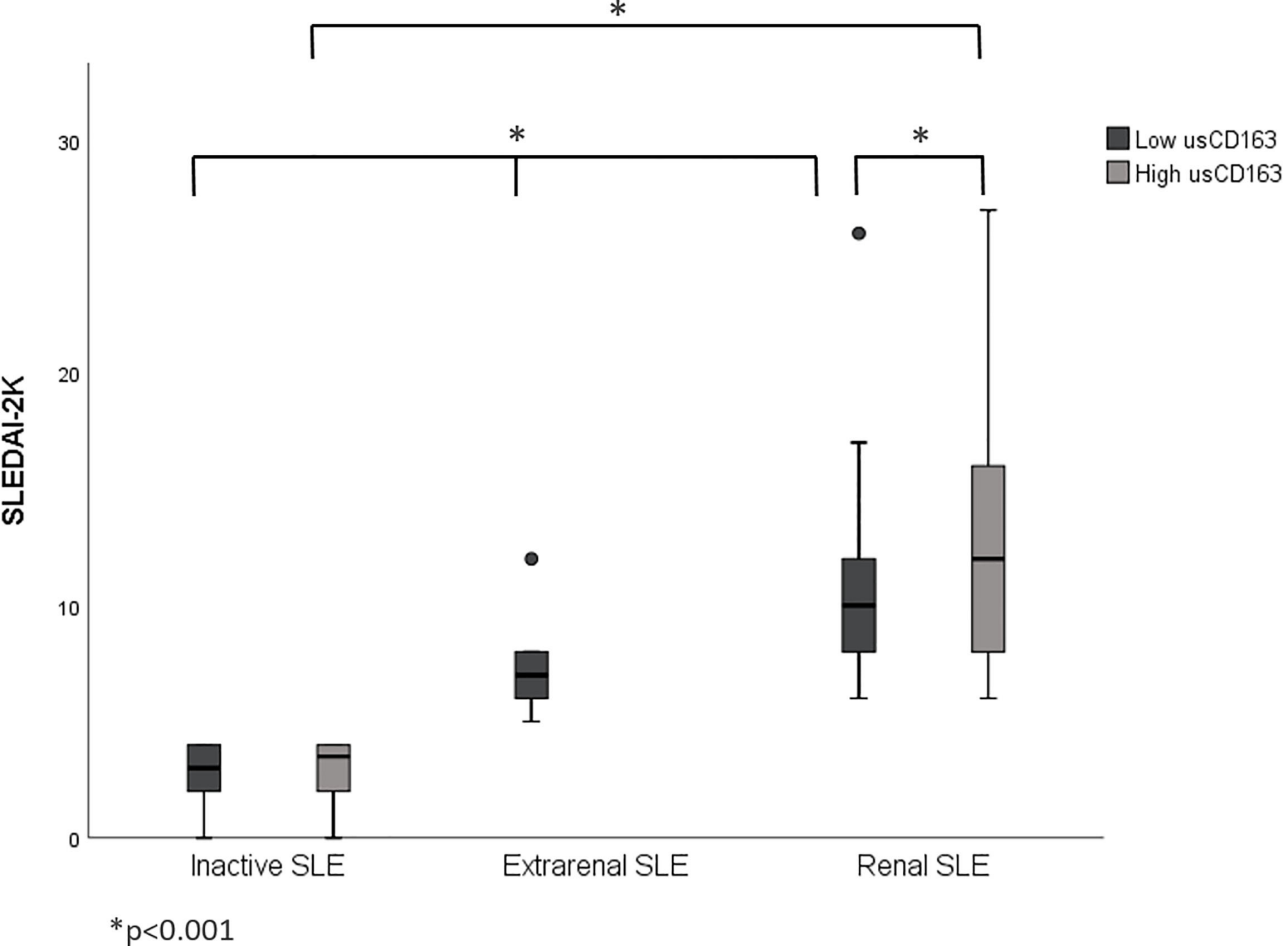
Correlation between usCD163 and SLEDAI-2K in SLE patients. The renal SLE patients with high usCD163 levels had higher SLEDAI-2K scores compared with those with low usCD163 levels. However, no difference in SLEDAI-2K score was seen between the inactive and extrarenal SLE patients. SLEDAI-2k, Systemic Lupus Erythematosus Disease Activity Index 2000.

**Figure 2:**
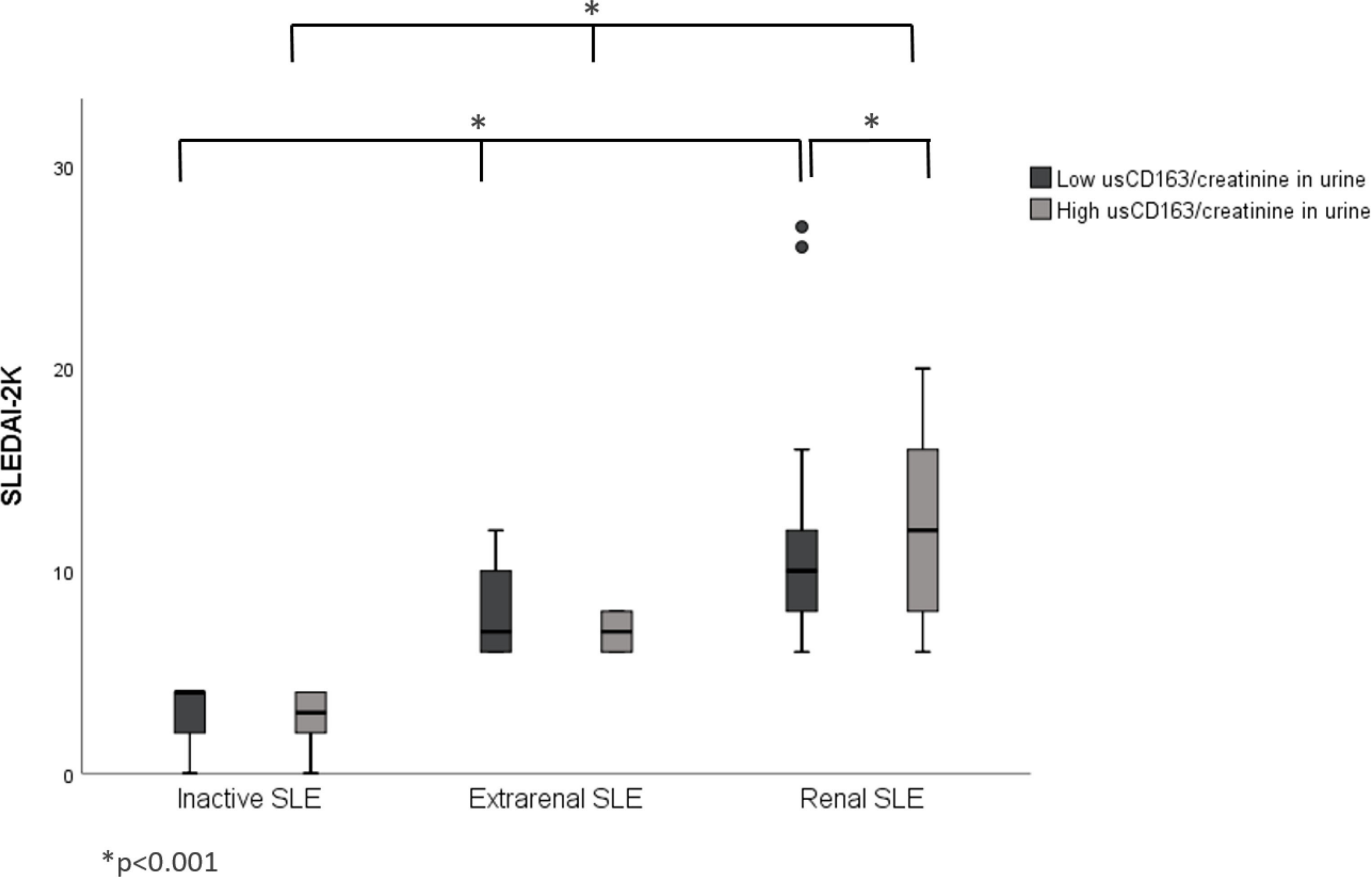
Correlation between usCD163/creatinine in urine and SLEDAI-2K in SLE patients. The renal SLE patients with high usCD163/creatinine ratios had higher SLEDAI-2K scores compared with those with low usCD163 levels. However, no difference in SLEDAI-2K score was seen between the inactive and extrarenal SLE patients. SLEDAI-2k, Systemic Lupus Erythematosus Disease Activity Index 2000.

The renal SLE patients with high usCD163 levels or usCD163/creatinine ratios had higher SLEDAI-2K scores compared with those with low usCD163 levels (**Figures 1**, **2**). However, no difference in SLEDAI-2K score was seen between the inactive and extrarenal SLE patients. The clinical characteristics of the inactive, extrarenal, and renal SLE patients are shown in **Supplementary Table S1**.

### Chronic Kidney Disease Stage of Patients With High and Low usCD163 Levels

**Figure 3:**
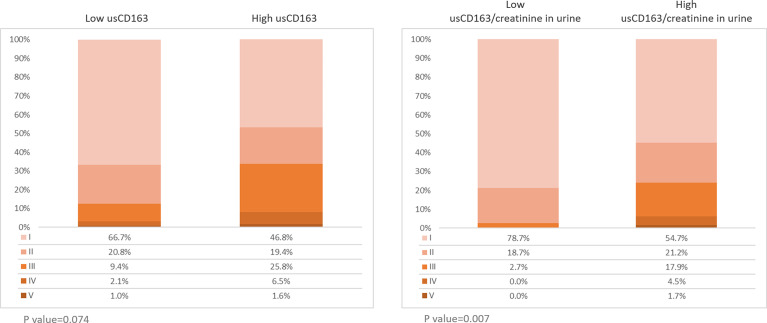
Stages of chronic kidney diseases in SLE patients. Two hundred sixty-one patients with SLE were divided into low or high usCD163 and usCD163/creatinine in urine groups. The stages of CKD were divided into stages I (EGFR≧90), II (EGFR 60-89), III (EGFR 30-59), IV (EGFR 15-29), and V (EGFR≦15).

The relative proportions of chronic kidney disease (CKD) stages I and II were higher in the low than high usCD163 group. The relative proportions of CKD stages II–V were higher in the high than low usCD163 group (**Figure 3**). SLE patients with high usCD163 levels tended to have a higher CKD stage.

## Discussion

Macrophages have been implicated in the pathogenesis of SLE, including classically activated inflammatory M1 macrophages and the alternately activated M2 macrophages M2a, M2b, and M2c, which demonstrate pro-fibrotic, immune-regulatory, remodeling, and anti-inflammatory effects, respectively. Initially, M1 macrophages were reported to be predominant in SLE, with M2a and M2c macrophage expressions being reduced (24). However, Gregor et al. detected more M2c than M2a macrophages, and few M1 macrophages, in all ISN/RPS classes of LN (25). Several reports suggest that M2 macrophages play an important role in driving or regulating interstitial inflammation, cellular crescent formation, and fibrinoid necrosis (9, 26, 27). These studies suggested that CD163+ M2c-like macrophages may be associated with the pathogenesis of SLE.

UsCD163 is a marker of the status of LN and could also serve as a biomarker of SLE disease activity. Mejia-Vilet et al. reported an association between usCD163 and proteinuria (9). Zhang et al. reported that the usCD163 level was correlated with the SLEDAI, rSLEDAI, and Physician Global Assessment scores in various ethnic groups (28). Our results showed that the usCD163 level and urine usCD163/creatinine ratio were correlated with the UPCR, anti-dsDNA Ab level, and SLEDAI and rSLEDAI scores. Furthermore, the usCD163 level of 0.443 ng/ml and the urine usCD163/creatinine ratio of 110.2 ng/mmol predicted high SLEDAI scores. Therefore, usCD163 might be considered as a marker to measure the disease activity of systemic lupus erythematosus or lupus nephritis in the future.

C3, C4, and anti-ds DNA Ab are traditional biomarkers of SLE disease activity; the SLEDAI-2K score is also based on these markers. We found that the usCD163 level and usCD163/creatinine ratio were also correlated with the SLEDAI-2K score and anti-ds DNA Ab level. Serum-soluble CD163 (29), monocyte chemoattractant protein-1 (MCP-1), neutrophil gelatinase-associated lipocalin (NGAL), TNF-like weak inducer of apoptosis (TWEAK), and vascular cell adhesion molecule-1 (VCAM) are new biomarkers of SLE disease activity (30).

Renal biopsy is the gold standard to diagnose LN. The identification of non-invasive biomarkers, such as usCD163, of active LN that are strongly correlated with renal biopsy results and underlying disease mechanisms has been a priority. Other non-invasive biomarkers of LN include anti-C1q antibody and urine MCP-1 (18, 31). The treatment goal should be an at least 25% and 50% reduction in the proteinuria level after 3 and 6 months, respectively, and a complete renal response (500–700 mg/day) at 12 months (32). However, persistent proteinuria (lasting for 6 months) makes it difficult to titrate immunosuppressants. Mejia-Vilet et al. found that the level of usCD163 after treatment could be monitored to predict the renal response and kidney histology score CD163 > 370 ng/mmol perfectly agreed (k = 1.0) with a histologic activity index > 1 in repeated biopsies after lupus nephritis flare-up (9).

The *in vivo* expression of CD163 on macrophages is influenced by several medications including glucocorticoids, mycophenolate mofetil (MMF), tacrolimus, rituximab, and cyclophosphamide (25, 33–36). However, the observed elevation in urine sCD163 in active and proliferative LN was not attributed to medications such as MMF and glucocorticoids in clinical studies (4, 28).

In our study, we found that mycophenolate sodium was more likely in patients with high usCD163. Actually, mycophenolate sodium and mycophenolate mofetil are all mycophenolate acids (MPAs) (37). Unlike oral mycophenolate mofetil, which releases MPA in the stomach, enteric-coated mycophenolate sodium releases MPA in the small intestine. Renal function, serum albumin levels, sex, ethnicity, food, concurrent antacids, metal-containing medications, and proton pump inhibitors were identified in some studies as having a significant influence on the pharmacokinetics of mycophenolate (38). The reason why patients with mycophenolate sodium tend to have high usCD163 is still unknown.

As a biomarker, usCD163 can be evaluated non-invasively and directly reflects macrophage-mediated glomerular inflammation; it can also distinguish between class III/IV and V LN in patients showing proteinuria relapse during their clinical course (4, 9, 28). The reason why usCD163 correlates with the proteinuria level and UPCR, but not with class V LN, needs further study.

The studies about the correlation of serum CD163 with systemic lupus erythematosus were conflicting. Serum CD163 was reported to be a biomarker that could be measured in lupus nephritis, accelerated atherosclerosis, and macrophage activation syndrome of SLE (39–41). On the other hand, Nobuhide et al. pointed out that serum CD163 levels did not show a difference between lupus nephritis, ANCA-associated vasculitis, and diabetic nephropathy (4). Moreover, there was a weak association between serum CD163 concentration and histological activity in lupus nephritis.

One of novel points in our study is that we compared both usCD163 and usCD163/creatinine in urine in systemic lupus erythematosus patients. Next, we analyzed the CKD stages in SLE patients with high and low usCD163 that was not discussed by other papers. Third, we found out that the patients with mycophenolate sodium but not mycophenolate mofetil might have a higher usCD163 level that was not mentioned by others.

The first limitation of our study was that patients with lupus nephritis did not receive a renal biopsy (53/96) for several reasons including low lupus nephritis disease activity, contraindication to renal biopsy, or lupus nephritis easily controlled by conventional disease-modifying antirheumatic drugs. Besides, the initial renal histopathology cannot represent the subsequent disease activity of lupus nephritis during the follow-up period. However, a previous study showed that usCD163 was associated with proliferative LN classes III and IV. Second, serial usCD163 measurements and long-term follow-up of renal were not performed, as this was a cross-sectional study. We will further analyze serum CD163, usCD163, anti-C1q antibody, and urine MCP-1 levels in LN in future studies. Lastly, we did not include a matched healthy control population in our study.

## Conclusion

Most of our patients with a high usCD163 level or usCD163/creatinine ratio were renal SLE patients and had high SLEDAI and rSLEDAI scores. The usCD163 level and usCD163/creatinine ratio correlated with the UPCR, anti-ds DNA level, and SLEDAI and rSLEDAI scores. The strong correlation between renal SLE and disease activity indicates that further study of usCD163 is merited, as a promising biomarker of LN.

## Data Availability Statement

The original contributions presented in the study are included in the article/**Supplementary Material**. Further inquiries can be directed to the corresponding author.

## Ethics Statement

The studies involving human participants were reviewed and approved by Institutional Review Board of Chang Gung Memorial Hospital. The patients/participants provided their written informed consent to participate in this study. Written informed consent was obtained from the individual(s) for the publication of any potentially identifiable images or data included in this article.

## Author Contributions

Conceptualization, C-FK; methodology, Y-JH and C-HL; software, Y-JH and C-HL; validation, C-FK; formal analysis, YJH and C-HL; investigation, Y-JH; resources, H-YY and C-FK; data curation, Y-JH and C-HL; writing—original draft preparation, Y-JH; writing—review and editing, Y-JH and CFK; visualization, Y-JH; supervision, S-FL; project administration, C-FK and S-FL; funding acquisition, C-FK. All authors have read and agreed to the published version of the manuscript.

## Funding

This study received funding from Key Development Project of Department of Science and Technology (2015C03Bd051) and Chang Gung Memorial Hospital Research Program (CMRPG3J0031).

## Conflict of Interest

The authors declare that the research was conducted in the absence of any commercial or financial relationships that could be construed as a potential conflict of interest.

## Publisher’s Note

All claims expressed in this article are solely those of the authors and do not necessarily represent those of their affiliated organizations, or those of the publisher, the editors and the reviewers. Any product that may be evaluated in this article, or claim that may be made by its manufacturer, is not guaranteed or endorsed by the publisher.

## References

[1] Møller HJ. Soluble Cd163. Scand J Clin Lab Invest (2012) 72(1):1–13. doi: 10.3109/00365513.2011.626868 22060747

[2] Etzerodt A Moestrup SK. CD163 and Inflammation: Biological, Diagnostic, and Therapeutic Aspects. Antioxid Redox Signal (2013) 18(17):2352–63. doi: 10.1089/ars.2012.4834 PMC3638564 22900885

[3] Murray PJ. Macrophage Polarization. Annu Rev Physiol (2017) 79:541–66. doi: 10.1146/annurev-physiol-022516-034339 27813830

[4] Endo N Tsuboi N Furuhashi K Shi Y Du Q Abe T. Urinary Soluble CD163 Level Reflects Glomerular Inflammation in Human Lupus Nephritis. Nephrol Dial Transplant (2016) 31(12):2023–33. doi: 10.1093/ndt/gfw214 27242373

[5] Arai M Mii A Kashiwagi T Shimizu A Sakai Y. The Severity of Glomerular Endothelial Cell Injury is Associated With Infiltrating Macrophage Heterogeneity in Endocapillary Proliferative Glomerulonephritis. Sci Rep (2021) 11(1):13339. doi: 10.1038/s41598-021-92655-5 34172770 PMC8233400

[6] Zhou D Wang Y Chen LU Zhang W Luan J. Soluble CD163: A Novel Biomarker With Diagnostic and Therapeutic Implications in Autoimmune Diseases. J Rheumatol (2016) 43(4):830. doi: 10.3899/jrheum.151317 27037247

[7] Van Gorp H Delputte PL Nauwynck HJ. Scavenger Receptor CD163, a Jack-Of-All-Trades and Potential Target for Cell-Directed Therapy. Mol Immunol (2010) 47(7-8):1650–60. doi: 10.1016/j.molimm.2010.02.008 20299103

[8] Matsushita T Takehara K. Soluble CD163 Is a Potential Biomarker in Systemic Sclerosis. Expert Rev Mol Diagn (2019) 19(3):197–9. doi: 10.1080/14737159.2019.1571911 30657715

[9] Mejia-Vilet JM Zhang XL Cruz C Cano-Verduzco ML Shapiro JP Nagaraja HN. Urinary Soluble CD163: A Novel Noninvasive Biomarker of Activity for Lupus Nephritis. J Am Soc Nephrol (2020) 31(6):1335–47. doi: 10.1681/ASN.2019121285 PMC7269356 32300067

[10] Yang H Liu H Zhou Z Zhao L Fei Y Chen H. Management of Severe Refractory Systemic Lupus Erythematosus: Real-World Experience and Literature Review. Clin Rev Allergy Immunol (2021) 60(1):17–30. doi: 10.1007/s12016-020-08817-2 33159635

[11] Ichinose K. [Unmet Needs in Systemic Lupus Erythematosus]. Nihon Rinsho Meneki Gakkai Kaishi (2017) 40(6):396–407. doi: 10.2177/jsci.40.396 29367524

[12] Mohan C Putterman C. Genetics and Pathogenesis of Systemic Lupus Erythematosus and Lupus Nephritis. Nat Rev Nephrol (2015) 11(6):329–41. doi: 10.1038/nrneph.2015.33 25825084

[13] Bajema IM Wilhelmus S Alpers CE Bruijn JA Colvin RB Cook HT. Revision of the International Society of Nephrology/Renal Pathology Society Classification for Lupus Nephritis: Clarification of Definitions, and Modified National Institutes of Health Activity and Chronicity Indices. Kidney Int (2018) 93(4):789–96. doi: 10.1016/j.kint.2017.11.023 29459092

[14] Tektonidou MG Dasgupta A Ward MM. Risk of End-Stage Renal Disease in Patients With Lupus Nephritis, 1971-2015: A Systematic Review and Bayesian Meta-Analysis. Arthritis Rheumatol (2016) 68(6):1432–41. doi: 10.1002/art.39594 PMC5071782 26815601

[15] Tofighi T Morand EF Touma Z. Systemic Lupus Erythematosus Outcome Measures for Systemic Lupus Erythematosus Clinical Trials. Rheum Dis Clin North Am (2021) 47(3):415–26. doi: 10.1016/j.rdc.2021.04.007 34215371

[16] Arriens C Wren JD Munroe ME Mohan C. Systemic Lupus Erythematosus Biomarkers: The Challenging Quest. Rheumatol (Oxford) (2017) 56(suppl_1):i32–45. doi: 10.1093/rheumatology/kew407 PMC5850341 28013203

[17] Hull KL Adenwalla SF Topham P Graham-Brown MP. Indications and Considerations for Kidney Biopsy: An Overview of Clinical Considerations for the non-Specialist. Clin Med (Lond) (2021) 22:34–40. doi: 10.7861/clinmed.2021-0472 PMC8813013 34921054

[18] Rodríguez-Almaraz E Gutiérrez-Solís E Rabadán E Rodríguez P Carmona L Morales E. Something New About Prognostic Factors for Lupus Nephritis? A Systematic Review. Lupus (2021) 30:9612033211061475. doi: 10.1177/09612033211061475 34907831

[19] Radin M Miraglia P Barinotti A Fenoglio R Roccatello D Sciascia S. Prognostic and Diagnostic Values of Novel Serum and Urine Biomarkers in Lupus Nephritis: A Systematic Review. Am J Nephrol (2021) 52(7):559–71. doi: 10.1159/000517852 34515043

[20] Aragón CC Tafúr RA Suárez-Avellaneda A Martínez T de Las Salas A Tobón GJ. Urinary Biomarkers in Lupus Nephritis. J Transl Autoimmun (2020) 3:100042. doi: 10.1016/j.jtauto.2020.100042 32743523 PMC7388339

[21] Tan EM Cohen AS Fries JF Masi AT McShane DJ Rothfield NF. The 1982 Revised Criteria for the Classification of Systemic Lupus Erythematosus. Arthritis Rheum (1982) 25(11):1271–7. doi: 10.1002/art.1780251101 7138600

[22] Petri M Orbai AM Alarcón GS Gordon C Merrill JT Fortin PR. Derivation and Validation of the Systemic Lupus International Collaborating Clinics Classification Criteria for Systemic Lupus Erythematosus. Arthritis Rheum (2012) 64(8):2677–86. doi: 10.1002/art.34473 PMC3409311 22553077

[23] Gladman DD Ibañez D Urowitz MB. Systemic Lupus Erythematosus Disease Activity Index 2000. J Rheumatol (2002) 29(2):288–91. 11838846

[24] Orme J Mohan C. Macrophage Subpopulations in Systemic Lupus Erythematosus. Discovery Med (2012) 13(69):151–8. 22369974

[25] Olmes G Büttner-Herold M Ferrazzi F Distel L Amann K Daniel C. CD163+ M2c-Like Macrophages Predominate in Renal Biopsies From Patients With Lupus Nephritis. Arthritis Res Ther (2016) 18:90. doi: 10.1186/s13075-016-0989-y 27091114 PMC4835936

[26] Tao J Zhao J Qi XM Wu YG. Complement-Mediated M2/M1 Macrophage Polarization may be Involved in Crescent Formation in Lupus Nephritis. Int Immunopharmacol (2021) 101(Pt A):108278. doi: 10.1016/j.intimp.2021.108278 34700131

[27] Gupta R Yadav A Aggarwal A. Urinary Soluble CD163 Is a Good Biomarker for Renal Disease Activity in Lupus Nephritis. Clin Rheumatol (2021) 40(3):941–8. doi: 10.1007/s10067-020-05343-6 32809146

[28] Zhang T Li H Vanarsa K Gidley G Mok CC Petri M. Association of Urine Scd163 With Proliferative Lupus Nephritis, Fibrinoid Necrosis, Cellular Crescents and Intrarenal M2 Macrophages. Front Immunol (2020) 11. doi: 10.3389/fimmu.2020.00671 PMC7174755 32351512

[29] Lindblom J Mohan C Parodis I. Diagnostic, Predictive and Prognostic Biomarkers in Systemic Lupus Erythematosus: Current Insights. Curr Opin Rheumatol (2022) 34(2):139–49. doi: 10.1097/BOR.0000000000000862 35013077

[30] Soliman S Mohan C. Lupus Nephritis Biomarkers. Clin Immunol (2017) 185:10–20. doi: 10.1016/j.clim.2016.08.001 27498110

[31] Dias R Hasparyk UG Lopes MP de Barros JLVM Silva ACSE. Novel Biomarkers for Lupus Nephritis in the "OMICS" Era. Curr Med Chem (2021) 28(29):6011–44. doi: 10.2174/0929867328666210212102438 33583367

[32] Kostopoulou M Fanouriakis A Cheema K Boletis J Bertsias G Jayne D. Management of Lupus Nephritis: A Systematic Literature Review Informing the 2019 Update of the Joint EULAR and European Renal Association-European Dialysis and Transplant Association (EULAR/ERA-EDTA) Recommendations. RMD Open (2020) 6(2):713–23. doi: 10.1136/rmdopen-2020-001263 PMC7425195 32699043

[33] Tang Z Niven-Fairchild T Tadesse S Norwitz ER Buhimschi CS Buhimschi IA. Glucocorticoids Enhance CD163 Expression in Placental Hofbauer Cells. Endocrinology (2013) 154(1):471–82. doi: 10.1210/en.2012-1575 PMC3529384 23142809

[34] Kannegieter NM Hesselink DA Dieterich M Kraaijeveld R Rowshani AT Leenen PJM. The Effect of Tacrolimus and Mycophenolic Acid on CD14+ Monocyte Activation and Function. PloS One (2017) 12(1):e0170806. doi: 10.1371/journal.pone.0170806 28122021 PMC5266297

[35] Guo ZS Parimi V O'Malley ME Thirunavukarasu P Sathaiah M Austin F. The Combination of Immunosuppression and Carrier Cells Significantly Enhances the Efficacy of Oncolytic Poxvirus in the Pre-Immunized Host. Gene Ther (2010) 17(12):1465–75. doi: 10.1038/gt.2010.104 PMC2982886 20703311

[36] Lapuc I Bolkun L Eljaszewicz A Rusak M Luksza E Singh P. Circulating Classical CD14++CD16- Monocytes Predict Shorter Time to Initial Treatment in Chronic Lymphocytic Leukemia Patients: Differential Effects of Immune Chemotherapy on Monocyte-Related Membrane and Soluble Forms of CD163. Oncol Rep (2015) 34(3):1269–78. doi: 10.3892/or.2015.4088 26135617

[37] Tett SE Saint-Marcoux F Staatz CE Brunet M Vinks AA Miura M. Mycophenolate, Clinical Pharmacokinetics, Formulations, and Methods for Assessing Drug Exposure. Transplant Rev (Orlando) (2011) 25(2):47–57. doi: 10.1016/j.trre.2010.06.001 21190834

[38] Abd Rahman AN Tett SE Staatz CE. Clinical Pharmacokinetics and Pharmacodynamics of Mycophenolate in Patients With Autoimmune Disease. Clin Pharmacokinet (2013) 52(5):303–31. doi: 10.1007/s40262-013-0039-8 23475567

[39] Nishino A Katsumata Y Kawasumi H Hirahara S Kawaguchi Y Yamanaka H. Usefulness of Soluble CD163 as a Biomarker for Macrophage Activation Syndrome Associated With Systemic Lupus Erythematosus. Lupus (2019) 28(8):986–94. doi: 10.1177/0961203319860201 31246559

[40] David C Divard G Abbas R Escoubet B Chezel J Chauveheid MP. Soluble CD163 is a Biomarker for Accelerated Atherosclerosis in Systemic Lupus Erythematosus Patients at Apparent Low Risk for Cardiovascular Disease. Scand J Rheumatol (2020) 49(1):33–7. doi: 10.1080/03009742.2019.1614213 31161842

[41] Yang G Guo N Yin J Wu J. Elevated Soluble CD163 Predicts Renal Function Deterioration in Lupus Nephritis: A Cohort Study in Eastern China. J Int Med Res (2021) 49(11):3000605211049963. doi: 10.1177/03000605211049963 34755559 PMC8586176

