# Corrigendum: Urine soluble CD163 is a promising biomarker for the diagnosis and evaluation of lupus nephritis

**DOI:** 10.3389/fimmu.2022.1003761

**Published:** 2022-08-29

**Authors:** Yun-Ju Huang, Chiung-Hung Lin, Huang-Yu Yang, Shue-Fen Luo, Chang-Fu Kuo

**Affiliations:** ^1^ School of Medicine, Chang Gung University, Taoyuan City, Taiwan; ^2^ Division of Rheumatology, Allergy and Immunology, Chang Gung Memorial Hospital, Taoyuan City, Taiwan; ^3^ Division of Thoracic medicine, Chang Gung Memorial Hospital, Taoyuan City, Taiwan; ^4^ Division of Nephrology, Allergy and Immunology, Chang Gung Memorial Hospital, Taoyuan City, Taiwan; ^5^ Center for Artificial Intelligence in Medicine, Chang Gung Memorial Hospital, Taoyuan, Taiwan

**Keywords:** Systemic lupus erythematosus, lupus nephritis, urine soluble CD163, urine biomarker, chronic kidney disease, macrophage, SLEDA

In the published article, there was an error in **Figure 2** as published. We mistakenly used **Figure 1** as **Figure 2**. The **Figure 1** describes us CD163 in urine and SLEDAI-2K in SLE patients while the **Figure 2** describes usCD163/creatinine in urine and SLEDAI-2K in SLE patients. The corrected **Figure 2** and its caption appear below.

**Figure 2:**
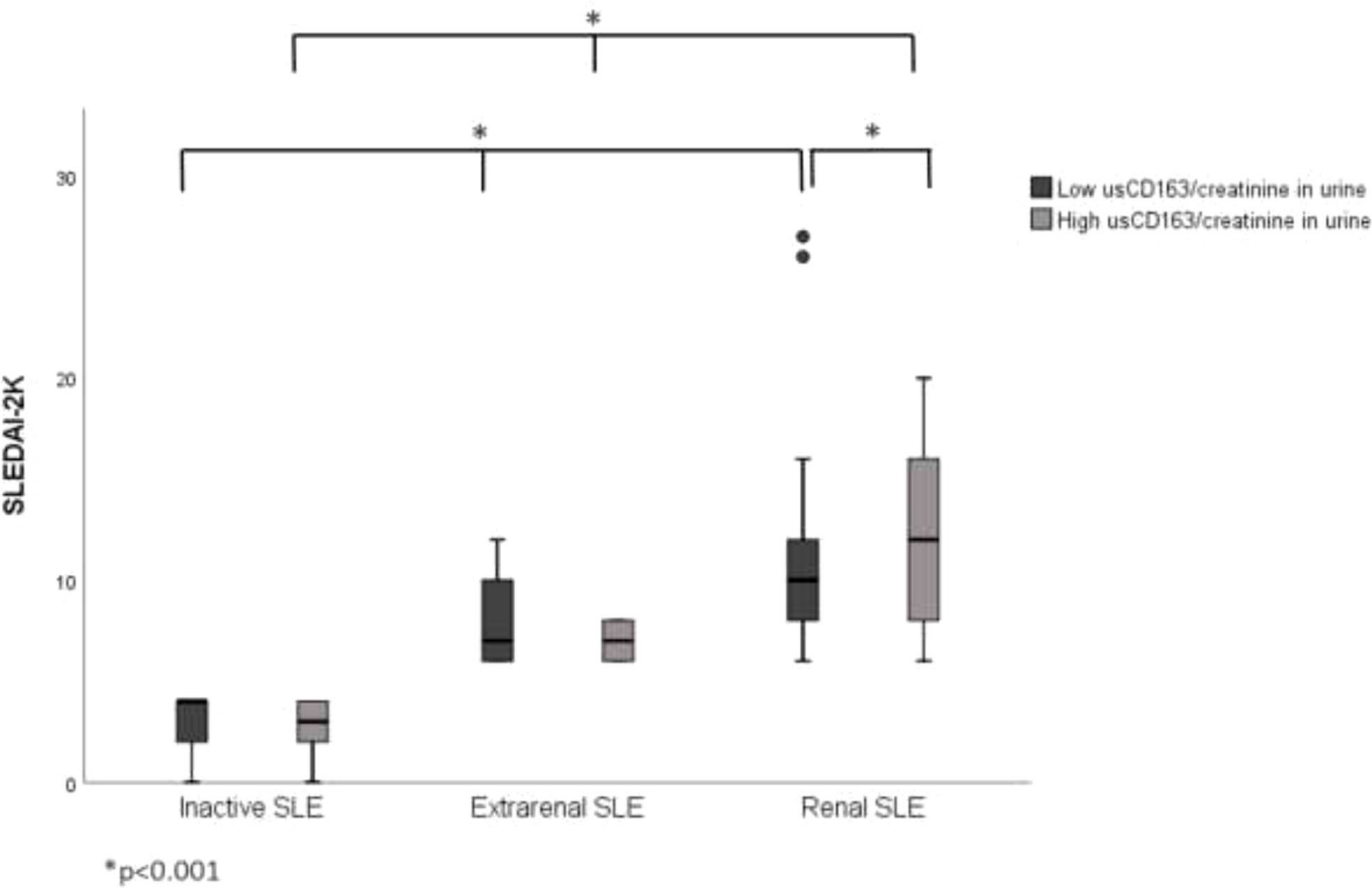
Correlation between usCD163/creatinine in urine and SLEDAI-2K in SLE patients. The renal SLE patients with high usCD163/creatinine ratios had higher SLEDAI-2K scores compared with those with low usCD163 levels. However, no difference in SLEDAI-2K score was seen between the inactive and extrarenal SLE patients. SLEDAI-2k, Systemic Lupus Erythematosus Disease Activity Index 2000.

In the published article, there was an error in the Funding statement. Funding from Chang Gung Memorial Hospital Research Program (CMRPG3J0031) was omitted. The correct Funding statement appears below.

## Funding

This study received funding from Key Development Project of Department of Science and Technology (2015C03Bd051) and Chang Gung Memorial Hospital Research Program (CMRPG3J0031).

The authors apologize for these errors and state that they do not change the scientific conclusions of the article in any way. The original article has been updated.

## Publisher’s note

All claims expressed in this article are solely those of the authors and do not necessarily represent those of their affiliated organizations, or those of the publisher, the editors and the reviewers. Any product that may be evaluated in this article, or claim that may be made by its manufacturer, is not guaranteed or endorsed by the publisher.

