# Supplementary material for: Urine Soluble CD163 Is a Promising Biomarker for the Diagnosis and Evaluation of Lupus Nephritis
Source: Front Immunol. 2022 Jul 14;13:935700. doi: 10.3389/fimmu.2022.935700 (PMC9329951; doi:10.3389/fimmu.2022.935700)
Supplement: Supplementary file 1 [file DataSheet_1.docx]

**Supplementary Table S1. Clinical and laboratory of inactive SLE, active non-renal SLE, active LN**

|  | Inactive SLE, n=96 | Extrarenal SLE, n=9 | Active LN, n=156 | P-value |
| --- | --- | --- | --- | --- |
| Age(years) | 47.1±11.3 | 39.4±6.4 | 43±12.1 | **0.012*** |
| Sex(male/female) | 8/88 | 0/9 | 4/152 | 0.084 |
| BMI(kg/m^2^) | 23.5±5(57.9,14) | 20.6±2.5(24.8,17.5) | 22.9±4.3(48.3,14.7) | 0.162 |
| SLEDAI-2K, points | 2.7±1.5(4,0) | 7.4±2.2(12,5) | 10.9±3.7(27,6) | **<0.001*** |
| Diabetes mellitus, n(%) | 7(7.3%) | 0(0%) | 7(4.5%) | **0.036*** |
| Admission, n(%) | 5(5.2%) | 0(0%) | 19(12.2%) | 0.111 |
| Macrophage activation syndrome, n(%) | 0 | 0 | 2(1.3%) | 0.507 |
| CNS lupus, n(%) | 0 | 0 | 1(0.6%) | 0.664 |
| Infection, n(%) | 2(2.1%) | 0(0%) | 6(3.8%) | 0.632 |
| Pleural effusion, n(%) | 0 | 1(11.1%) | 9(5.8%) | **0.035*** |
| Pericardial effusion, n(%) | 0 | 1(11.1%) | 3(1.9%) | **0.046*** |
| Laboratory involvement |  |  |  |  |
| Serum creatinine (mg/dL) | 0.8±0.9(7,0.2) | 0.1±0.1(0.9,0.5) | 0.8±0.5(4.2,0.3) | 0.728 |
| BUN (mmol/L) | 26.2±21.4(79,8) | 14.9±5.8(19,11) | 20.5±44.3(297,5) | 0.699 |
| EGFR (mL/min/1.73 m^2^) | 104.3±47.5(391,6) | 111.3±25.2(154,80) | 99±49.7(289,8) | 0.611 |
| UPCR (mg protein/g creatinine) | 134.6±277.1(2074,0) | 150.7±101.5(312,0) | 1414.6±2558.2(18393,0) | **<0.001*** |
| Semi-quantitative hematuria in urinary dipstick | None:83(88.3%)  Trace: 4(4.3%)  1+:5(5.3%)  2+:2(2.1%)  3+:0(0%) | None:7(77.8%)  Trace:0(0%)  1+:0(0%)  2+:0(0%)  3+:0(0%) | None:77(49.4%%)  Trace: 14(9%)  1+:23(12.8%)  2+:24(15.4%)  3+:21(13.5%) | **<0.001*** |
| Semi-quantitative proteinuria in urinary dipstick | None:75(79.8%)  Trace: 5(5.3%)  1+:9(9.6%)  2+:4(4.3%)  3+:1(1.1%)  4+:0(0%) | None:7(77.8%)  Trace: 0(0%)  1+:0(0%)  2+:0(0%)  3+:0(0%)  4+:0(0%) | None:60(38.5%)  Trace:20(11.2%)  1+:20(11.5%)  2+:24(17.3%)  3+:21(16%)  4+:8(5.1%) | **<0.001*** |
| RBC in urinary dipstick(HPF) | 3.6±14.7(3,0) | 0.9±1.5(500,0) | 34±83.8(500,0) | **0.002*** |
| WBC in urinary dipstick(HPF) | 8±32.2(3,0) | 0.6±1.1(500,0) | 42.7±82.1(500,0) | **0.000*** |
| ESR(mm/hr) | 20.9±14.5(62,1) | 37±30.7(89,10) | 26.5±22.3(140,2) | **0.044*** |
| CRP (mg/L) | 2.2±3.4(24,0) | 3.4±4.9(12,0) | 6.6±13.5(82,0) | **0.021*** |
| WBC (/uL) | 5790±2208(13000,2000) | 6980±4901(18000,3000) | 6750±3207(20000,2000) | **0.042*** |
| Hb (g/dL) | 12.7±1.6(19,8) | 11.4±1.7(14,9) | 11.9±1.9(18,7) | **0.001*** |
| Neutrophil (/uL) | 3783.5±2125.4(12126,748) | 4792.9±3980.2(13725,1525) | 5156.9±3018.2(19000,918) | **0.001*** |
| Lymphocyte (/uL) | 1571.6±672.2(4572,361.2) | 1189.6±599.2(1980,183) | 1210±734.2(3860,78.3) | **0.001*** |
| Platelet (1000/uL) | 225.1±66.7(392,5) | 223.4±151.5(528,40) | 237.7±96.6(547,46) | 0.531 |
| C3 (mg/dL) | 89.6±18.8(138,30) | 61.5±10(71,41) | 77±26.4(173,18) | **<0.001*** |
| C4 (mg/dL) | 17.7±7.4(38,3) | 10.6±7.1(25,3) | 13.9±8.1(41,2) | **<0.001*** |
| Anti-ds DNA Ab (IU/mL) | 112.5±110.9(554,40) | 219.5±169.7(494,14) | 204.3±179(648,40) | **0.006*** |
| usCD163 (ng/mL) | 0.5±0.3(1.3,0.2) | 0.6±0.2(1.3,0) | 4.3±11.8(96.3,0.3) | **0.005*** |
| High usCD63, n(%) | 6(6.3%) | 0(0%) | 57(36.5%) | **<0.001*** |
| usCD163/creatinine in urine(ng/mmol) | 122.7±106.1(441.7,13.2) | 198.4±158.7(442.9,47.4) | 515±1081.1(6926.8,15.7) | **0.007*** |
| High usCD163/creatinine in urine, n(%) | 13(13.5%) | 2(22.2%) | 54(34.6%) | **0.005*** |
| Medications |  |  |  |  |
| Prednisolone, n(%) | 71(74%) | 6(66.7%) | 131(84%) | 0.097 |
|  | 5.6±7.8mg/day | 11.1±14.3mg/day | 7.9±7.3mg/day | **0.029*** |
| Hydroxychloroquine, n(%) | 62(64.6%) | 8(88.9%) | 112(71.8%) | 0.214 |
|  | 156.3±135.2mg/day(400,0) | 211.1±127mg/day(400,0) | 202.6±152.4mg/day(400,0) | **0.044*** |
| Mycophenolate mofetil, n(%) | 4(4.2%) | 0(0%) | 12(7.7%) | 0.388 |
| Mycophenolate sodium, n(%) | 11(11.5%) | 1(11.1%) | 45(28.8%) | **0.004*** |
| Azathioprine, n(%) | 10(10.4%) | 1(11.1%) | 9(5.8%) | 0.913 |
| Cyclosporine, n(%) | 1(1%) | 1(1.5%) | 9(5.6%) | 0.111 |
| Methotrexate, n(%) | 1(8.3%) | 0(0%) | 11(7.1%) | 0.068 |
| Pulse steroid, n(%) | 1(1%) | 0(0%) | 4(2.6%) | 0.633 |

BMI, body mass index; CNS, central nervous system; BUN, blood urea nitrogen; EGFR, estimated glomerular filtration rate; UPCR, urine protein creatinine ratio; RBC, red blood cell; WBC, white blood cell; CRP, C reactive protein; ESR, erythrocyte sedimentation rate; Hb, hemoglobin; C3, complement 3; C4, complement 4; Anti-ds DNA Ab, anti-double strand DNA antibody; SLEDAI-2k, Systemic Lupus Erythematosus Disease Activity Index 2000

**Supplementary Figure 1. ROC curves of usCD163 was generated to predict SLE patients with high SLEDAI-2k scores(≧6 points). The best cut-off point (Youden’s index) of usCD163 to predict SLE patients with high SLEDAI-2k scores(≧6 points) is 0.443 ng/mL with sensitivity 84% and specificity 53.1%.**


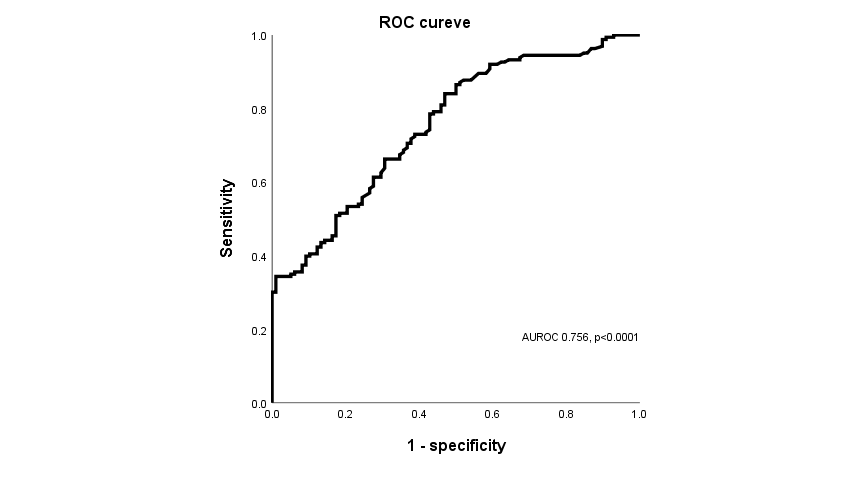


ROC, receiver operator characteristic; SLEDAI-2k, Systemic Lupus Erythematosus Disease Activity Index 2000

**Supplementary Figure 2. ROC curves of usCD163 was generated to predict SLE patients with high SLEDAI-2k scores(≧6 points). The best cut-off point (Youden’s index) of usCD163 to predict SLE patients with high SLEDAI-2k scores(≧6 points) is 110.2 ng/mmol with sensitivity 61% and specificity 68%.**


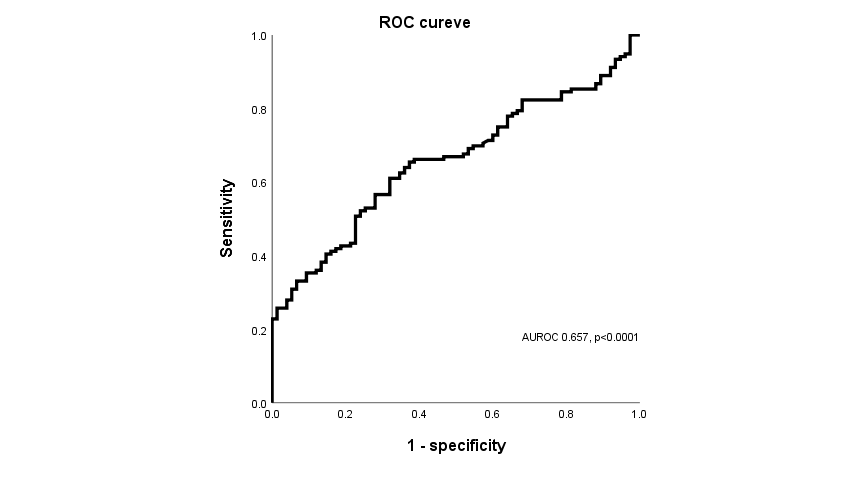


ROC, receiver operator characteristic; SLEDAI-2k, Systemic Lupus Erythematosus Disease Activity Index 2000
